# Clinicopathological Characteristics of Primary Appendiceal Mucinous Neoplasm and Recurrence After Radical Resection

**DOI:** 10.3389/fsurg.2022.902543

**Published:** 2022-05-04

**Authors:** Zaibiao Wang, Manman Yin, Jiayun Shao, Zhipeng Yin, Jie Peng, Zhengmao Lu

**Affiliations:** ^1^Department of General Surgery, Bozhou Hospital Affiliated to Anhui Medical University, Bozhou, China; ^2^Department of Science and Education, Bozhou Hospital Affiliated to Anhui Medical University, Bozhou, China; ^3^Department of Anesthesiology, Fudan University Shanghai Cancer Center, Shanghai, China; ^4^Department of Oncology, Shanghai Medical College, Fudan University, Shanghai, China; ^5^Department of General Surgery, Changhai Hospital, Naval Military Medical University of PLA, Shanghai, China

**Keywords:** appendiceal mucinous neoplasm, low-grade appendiceal mucinous neoplasm, PMP, rupture, non-LAMN

## Abstract

**Objective:**

Appendiceal mucinous neoplasm (AMN) is a rare obstructive dilatation of the appendix caused by an intraluminal accumulation of mucoid material, showing an insidious onset and few specific clinical manifestations. The purpose of the study is to analyze clinicopathological characteristics of primary AMN and recurrence after radical resection.

**Methods:**

A total of 50 patients were included in the retrospective cohort study of AMN. Patient data, such as demographics, tumor characteristics, surgical management, preoperative serum carcinoembryonic antigen (CEA), and carcinoembryonic antigen 19-9 (CA19-9) levels, were collected. All patients were followed-up with interval CT scans until the end of December 2021, with overall survival (OS) and progression-free survival (PFS) being calculated.

**Results:**

All patients were confirmed as AMN by pathological diagnosis after surgery, including 28 cases (56.00%) of low-grade AMN (LAMN) and 22 cases (44.00%) of non-LAMN. Among 50 patients with AMN, there were 12 cases (24.00%) complicated with pseudomyxoma peritonei (PMP). Higher proportions of patients with pTis, pT3, pT4a, ruptured at presentation, and PMP were found in patients with non-LAMN patients than LAMN (*p* < 0.05). There was a remarkable difference about preoperative serum CA19-9 levels between patients with LAMN and non-LAMN (*p* = 0.044). Patients complicated with PMP had a higher proportion of patients with ruptured at presentation than those who were not (*p* < 0.001). The patients with PMP had increased tumor size compared with those without PMP (*p* = 0.031). Remarkable differences were observed in terms of preoperative serum CA19-9 (*p* = 0.009) levels between patients with PMP and without PMP. We performed a multivariate analysis of the presence or absence of PMP and found that ruptured at presentation was found to be a risk factor for PMP in patients with AMN (*p* = 0.003). The PFS in the patients with PMP and those without was 33.33% (4/12) and 2.63% (1/38), showing a significant difference (*P* = 0.002).

**Conclusion:**

The study demonstrates that ruptured at presentation and PMP may influence the prognosis and survival of patients with AMN.

## Introduction

Appendectomy is a common surgical intervention. It is reported that about 1% of the appendectomy specimens are diagnosed with appendiceal tumors after pathological analysis (1), and appendiceal mucinous neoplasms (AMNs) are responsible for 0.2–0.3% of appendectomy specimens (2).

Appendiceal mucinous neoplasm is an uncommon disease and its prevalence is less than 1% of all cancers (3). Early criteria for simple mucocele considered AMN as a benign disease, also known as appendiceal mucocele (4), cystadenoma, and cystadenocarcinoma (5). The fifth edition of the World Health Organization (WHO) Classification of Tumors of the Digestive System divides AMN into low-grade and high-grade from morphological characteristics, such as structure, cytology, the presence of signet ring cells, and mitotic activity (6). Although this classification simplifies the diagnostic terminology of AMN, the ninth edition of the American Joint Committee on Cancer proposes descriptive terminology, such as well-differentiated (G1), moderately differentiated (G2), and poorly differentiated (G3), to classify AMN. Low-grade AMN (LAMN) is classified as G1, while high-grade AMN is classified as G2 or G3 (7). Studies have indicated that there are AMNs with relatively slow growth but high recurrence rate and high mortality, as well as AMNs with high invasiveness and an increased risk of early death (8, 9). AMN can lead to an increased risk of pseudomyxoma peritonei (PMP). LAMN with potential malignancy is significantly correlated with the presence of PMP. Furthermore, the increase of synchronous or metachronous colorectal cancer is associated with AMN (10, 11). PMP is a rare disease with a 1-per-million incidence rate (2), and is most frequently seen among women aged 50–70 years (12). PMP was first mentioned in the study of mucinous tumors of the ovary in 1884 (13). It is characterized by obvious diffuse intra-abdominal mucinous ascites on the surface of the peritoneum in clinical (14). This situation can be fatal if the patients with PMP are not treated.

Delayed diagnosis of appendiceal tumors often occurs due to their nonspecific clinical manifestations. In early-stage disease, the clinical manifestation presents pain in the right lower abdomen, commonly seen in acute appendicitis. A clinicopathological study of 184 patients with appendiceal tumors found that 32% of patients were misdiagnosed with acute appendicitis before the operation, and the proportion of patients accidentally diagnosed with appendiceal tumor was 23% (15).

Generally, these rare tumors are usually identified after surgery. More and more such tumors are diagnosed before any surgical operation, which benefits from the improvement of the availability and accuracy of medical imaging. The affecting factors, such as the stage at diagnosis and histological characteristics reflecting cell differentiation, are relevant to the clinical course of AMN (8, 9). In our retrospective report, we enrolled the clinical data of 50 patients with AMN and summarized the clinical characteristics of the disease and the factors affecting the prognosis, thus providing a reference for clinical diagnosis and treatment.

## Methods

### Patients and Data Collection

We retrospectively reviewed data of patients with a final pathological diagnosis of AMN who underwent primary surgical treatment at the Bozhou Hospital Affiliated with Anhui Medical University between January 2017 and October 2021. Those receiving neoadjuvant chemotherapy and those with a preoperative pathological diagnosis before the primary surgery were excluded from the retrospective analysis. The diagnosis of AMN was made according to the WHO classification and the Peritoneal Surface Oncology Group (PSOGI) consensus classification (16) with our routine practice by two pathologists. A third pathologist was invited to advise if there were significant differences between the two pathologists concerning the diagnosis, and we adopted the diagnosis of the third pathologist. We reviewed clinical information, physical examination notes, surgery, and pathological records of all included patients with the approval of the Institutional Review Board of the Bozhou Hospital Affiliated with Anhui Medical University.

### Biochemical Detection and Imaging Evaluation

Blood samples were collected from each patient before surgery for the detection of tumor biomarker, carcinoembryonic antigen (CEA), and carcinoembryonic antigen 19-9 (CA19-9). All patients had undergone at least one imaging evaluation (color Doppler ultrasound and CT).

### Variables

Patient data, such as demographics, tumor characteristics, and surgical management, were collected. Demographic variables included age, sex, and body mass index (BMI). Surgical modality included open or laparoscopic appendectomy. On pathological review, tumor characteristics included pT stage, tumor size, resection margin, and ruptured or non-ruptured appendix. Based on the American Joint Committee on Cancer (AJCC) Staging Standard (8th edition), the pT stage was classified into pTis, pT3, pT4a, and pT4b. pTis is defined as AMNs confined to the appendix (defined as involvement by acellular mucin or mucinous epithelium that may extend into the muscularis propria), pT3 is defined as tumor penetrating the muscularis propria or fibrotic appendix wall and invading the subserosa or mesoappendix, pT4a is defined as tumor perforating the visceral peritoneum, such as mucinous peritoneal tumors or acellular mucinous tumors on the serosa of the appendix or mesoappendix, and pT4b is defined as tumor directly invading other organs or structures. Tumor size was calculated according to either the pathology report, operative note, or radiological report of preoperative imaging. The ruptured status, which was defined as complete penetration of mucin from the lumen to the serosal surface with evidence of discontinuity of the appendiceal wall, was evaluated according to the final pathology report (17).

### Follow-Up

All patients were followed-up with interval CT scans until the end of December 2021. Overall survival (OS) was calculated from the date of surgery to the last date of follow-up or date of death. Progression-free survival (PFS) was calculated from the time of surgery to the date of disease progression confirmed by imaging motility.

### Statistical Analysis

Data processing and analysis were performed using SPSS software version 13.0 (IBM Inc., Armonk, NY, USA). Median and ranges were used to report continuous variables when non-normally distributed and mean ± standard deviation (SD) to report continuous variables when normally distributed. Frequencies and percentages were reported for categorical values. Multivariate analysis was employed to evaluate prognostic factors. A value of *p* < 0.05 reflects a statistically significant difference by the *t*-test, Mann–Whitney *U*-test, or the chi-square test.

## Results

### Patient Demographics

A total of 50 patients were included in the retrospective cohort study of AMN, among whom there were 19 men and 31 women. Patient age ranged from 21 to 81 years, with a mean age of (56.04 ± 17.13) years. There were 12 patients (24.00%) aged <44 years, 11 patients (22.00%) aged from 45 to 59 years, and 27 patients (54.00%) aged more than 60 years. The BMI of patients was 27.84 ± 5.35. The interval between the first symptoms and surgery of 50 patients was (25.5 [3.25, 120]) days.

### Clinical Characteristics of Patients With AMN

Among 50 patients, 15 cases (30.00%) showed acute abdominal diseases at the visit, such as right lower abdominal pain (*n* = 12), total abdominal pain (*n* = 2), and upper abdominal pain (*n* = 1); the majority of cases presented no acute abdominal disease at visit (70.00%). There were 13 cases (26%) that had a history of right lower abdominal mass. About preoperative detection of an inflammatory response, 9 cases (18.00%) of 50 patients were found to have increased white blood cell and neutrophil counts. All patients had undergone color Doppler ultrasound (*n* = 37) and/or CT (*n* = 29). Before surgery, 43 cases (86.00%) were diagnosed as AMN, while 5 cases were misdiagnosed as acute appendicitis due to no evident cystic lesion on the appendix or surrounding tissues on preoperative color Doppler ultrasound or CT and 2 cases as digestive tract perforation due to perihepatic gas effusion. There were 37 patients (74.00%) undergoing open appendectomy and 13 patients (26.00%) undergoing laparoscopic appendectomy. Ruptured at presentation was found in 10 patients. The tumor size was (6.85 ± 3.39) mm. All patients were confirmed as AMN by pathological diagnosis after surgery, including 34 cases (68.00%) of appendiceal mucocele, 14 cases (28.00%) of mucinous cystadenoma of the appendix, 2 cases (4%) of mucinous adenocarcinoma of appendix; 28 cases (56.00%) of LAMN and 22 cases (44.00%) of non-LAMN. Among 50 patients with AMN, there were 12 cases (24.00%) complicated with PMP.

### Surgical Management of Patients With AMN

All patients underwent complete surgical resection. During surgery, 20 patients (40.00%) who found their tumors in the middle-distal appendix were given appendectomy, 23 patients (46.00%) who had a whole appendix involved by the tumor or found their tumors at the end of the appendix were given appendectomy with partial cecectomy, and 7 patients (14.00%) who found their tumors involving the cecum and colon were given appendectomy with right hemicolectomy. Among 50 patients, 33 patients (66.00%) were defined as pTis stage, 10 patients (20.00%) were defined as pT3, and 7 patients (14.00%) were defined as pT4a; 5 patients (10.00%) had a positive resection margin and 45 patients (90.00%) had a negative resection margin. As for those with tumors ruptured at presentation, additional lymph node dissection was performed.

### Association Between Clinical Characteristics and LAMN

Demographics, surgical management, and tumor characteristics of patients stratified by LAMN and non-LAMN classification are listed in Table 1. No significant difference was found regarding age, gender distribution, BMI, surgical modality, tumor size, and preoperative serum CEA levels between patients with LAMN and non-LAMN (*p* > 0.05). Of note, there were remarkable differences in pTis, pT3, and pT4a between patients with LAMN and non-LAMN (*p* < 0.001; *p* = 0.015; and *p* = 0.035). A higher proportion of patients with ruptured at presentation was found in patients with non-LAMN than LAMN (*p* = 0.010). In addition, we found a higher proportion of patients complicated with PMP in patients with non-LAMN than LAMN (*p* = 0.013). There was a remarkable difference in preoperative serum CA19-9 levels between patients with LAMN and non-LAMN (*p* = 0.044). It was revealed that pTis, ruptured at presentation, the presence of PMP, and CA19-9 levels may contribute to the aggressiveness of AMN.

**Table 1:** Demographics, surgical management, tumor characteristics of patients stratified by LAMN and non-LAMN classification.

**Variables**	**LAMN (*n* = 28)**	**Non-LAMN (*n* = 22)**	***P***
Age (year)	55.05 ± 16.39	58.04 ± 17.94	0.542
Female, n (%)	18 (64.29%)	13 (59.09%)	0.774
BMI	27.83 ± 5.75	27.86 ± 4.94	0.985
Open appendectomy, *n* (%)	20 (71.43%)	17 (77.27%)	0.751
pT stage, *n* (%)			
pTis (*n* = 33)	25 (89.29%)	8 (36.36%)	<0.001
pT3 (*n* = 10)	2 (7.14%)	8 (36.36%)	0.015
pT4a (*n* = 7)	1 (3.57%)	6 (27.27%)	0.035
Ruptured at presentation, *n* (%)	2 (14.29%)	8 (27.27%)	0.010
Tumor size (mm)	6.55 ± 3.48	7.23 ± 3.32	0.488
PMP, *n* (%)	3 (17.86%)	9 (31.82%)	0.013
Preoperative serum CEA levels (ng/mL), median (range)	3.12 (0–438.59)	4.00 (0–2,536)	0.172
Preoperative serum CA19-9 levels (ng/mL), median (range)	9.34 (0–1,524.55)	15.31 (0–8824.33)	0.044

*Median and ranges were compared using Mann-Whitney test; Mean ± standard deviation were compared using t test. Frequencies and percentages were compared using chi-square test*.

### Risk Factors of PMP in Patients With AMN

Demographics, surgical management, and tumor characteristics of patients stratified by the presence and absence of PMP are shown in Table 2. It was found that the patients complicated with PMP were older than those who were not (*p* = 0.007). Patients complicated by PMP had a higher proportion of patients with ruptured at presentation than those who were not (*p* < 0.001). The patients with PMP had increased tumor size compared with those without (*p* = 0.031). Remarkable differences were observed in terms of preoperative serum CA19-9 (*p* = 0.009) levels between patients with PMP and without. There was no significant difference in gender distribution, age-stratification of 65-year-old BMI, surgical modality, and preoperative serum CEA levels between patients with PMP and without (*p* > 0.05). These observations suggested age, rupture at presentation, tumor size, and CA19-9 levels may contribute to the progression of PMP. We performed a multivariate analysis of the presence or absence of PMP by including age (assigned as actual values), ruptured at presentation (assigned as 1 = yes; 0 = no), and tumor size (assigned as actual values). It was revealed that ruptures at presentation were found as risk factors of PMP in patients with AMN (*p* = 0.003, Table 3).

**Table 2:** Demographics, surgical management, tumor characteristics of patients stratified by the presence and absence of PMP.

**Variables**	**Presence (*n* = 12)**	**Absence (*n* = 38)**	***P***
Age (year)	67.42 ± 11.00	52.45 ± 17.24	0.007
Age ≥ 65 years, *n* (%)	8 (66.67%)	14 (36.84%)	0.099
Female, *n* (%)	9 (75.00%)	22 (57.89%)	0.332
BMI	27.51 ± 5.02	27.95 ± 5.52	0.807
Open appendectomy, *n* (%)	9 (75.00%)	28 (73.68%)	0.928
Ruptured at presentation, *n* (%)	8 (66.67%)	1 (2.63%)	<0.001
Tumor size (mm)	8.38 ± 4.34	6.37 ± 2.94	0.031
Preoperative serum CEA levels (ng/mL), median (range)	7.98 (0–2,904.56)	2.42 (0–357.46)	0.208
Preoperative serum CA19-9 levels (ng/mL), median (range)	16.27 (0–9,264)	7.55 (0–1,280.43)	0.009

*Median and ranges were compared using Mann-Whitney test; Mean ± standard deviation were compared using t-test. Frequencies and percentages were compared using chi-square test*.

**Table 3:** Multivariate analysis of the presence or absence of PMP.

**Variables**	**Exp**	**95%CI**	***P***
Age (year)	1.087	0.992–1.191	0.075
Ruptured at presentation, n (%)	51.793	3.713–722.477	<0.001
Tumor size (mm)	1.124	0.826–1.530	0.455

### Follow-Up Analysis

All patients were followed-up with interval CT scans until the end of December 2021. The median follow-up time was 19 months, ranging from 2 to 47.5 months. The OS did not exhibit a significant difference between patients with non-LAMN and LAMN, between patients with PMP and without due to nobody dying. CT scans showed 4 patients with PMP who had been diagnosed with PMP and had tumors ruptured at a presentation during surgery. There was a case of strangulated hernia. The PFS was 90.00% (5/50). The PFS in the patients with non-LAMN and LAMN was 13.64% (3/22) and 7.14% (2/28), respectively, showing no significant difference (*p* > 0.05). The PFS in the patients with PMP and those without was 33.33% (4/12) and 2.63% (1/38), respectively, showing a significant difference (*p* = 0.002). These data indicate that PFS of patients with AMN is more closely associated with PMP than primary appendiceal pathological grade.

## Discussion

Appendiceal tumors are uncommon and often manifest as appendicitis, which is usually accidentally found in an acute situation (18). Neuroendocrine-related appendiceal tumors account for ~65% of histological classification of appendiceal tumors, and 20% of these tumors are adenocarcinoma which is defined as the most prevalent malignant histological subtype (19). In all subtypes, more than 50% of populations were subjective to adenocarcinoma (20). Most appendiceal tumors are not malignant and can be treated by appendectomy. AMN is a rare clinical disease, but the incidence rate is rising, accounting for 58% of all appendiceal tumors (21).

The nomenclature and diagnostic criteria of AMN have not been well identified for a long time. According to cytology, AMN is classified into low-grade and high-grade by the WHO Classification of Tumors of the Digestive System (2019), which is defined as a tumor with appendiceal mucinous epithelial hyperplasia, accompanied by extracellular mucus and “pushing” tumor border (6). Some studies demonstrated that KRAS and GNAS gene mutations occurred in LAMN (22, 23), and patients with high-grade AMN manifested mutations of KRAS and GNAS, along with TP53, as well as ATM (24). The pathogenesis needs to be further studied. At present, AMN is regarded as a disease of cystic expansion of the appendix caused by obstruction of the appendiceal cavity, leading to failure in the discharge of mucus secreted by the appendix mucosa. AMN can be classified into 4 types, such as retention cyst, epithelial hyperplasia cyst, mucinous cystadenoma, and mucinous cystadenocarcinoma, based on causes of obstruction of the appendix (25). It is difficult to judge whether AMN is benign or malignant simply. The initial affecting factors of AMN can be the same, but different evolution can occur, resulting in the existence of PMP (2) in the absence of hematogenic or lymphatic metastasis (26). Therefore, it is necessary to analyze AMN in combination with the biological characteristics of malignant tumors.

Appendiceal mucinous neoplasm exhibits non-specificity clinical features. In this study, 15 cases (30.00%) showed acute abdominal diseases, such as right lower abdominal pain (12 cases), total abdominal pain (2 cases), and upper abdominal pain (1 case). The majority of patients presented no acute abdominal disease (70.00%), suggesting that patients with an acute abdominal disease might be associated with AMN. In recent years, with the development of imaging technology, the accuracy of preoperative diagnosis of AMN has improved. AMN presents some ultrasound characteristics, such as location in the right lower abdomen, the anatomical position closing to the right psoas major muscle or iliac blood vessel, shaped like a tube or a circle, cystic or solid lesions, non-thick cystic wall, and the presence of calcification (27, 28). Ultrasound detection reveals a false negative rate to a certain extent due to the effect induced by intestinal gas. It is necessary to perform an abdominal CT examination for those with an unknown diagnosis. The CT examination assists in judging the correlation between the tumors and surrounding tissues, contributing to improvements in the preoperative diagnosis rate (29). In this study, all patients underwent abdominal color Doppler ultrasound or CT before surgery. We found that 43 cases (86.00%) were diagnosed with AMN before surgery. Then, 7 cases (14.00%) were misdiagnosed with other diseases, including acute appendicitis (5 cases) and digestive tract perforation caused by perihepatic gas effusion (2 cases). In the 5 patients with acute appendicitis, there was no evident cystic lesion on the appendix or surroundings before surgery.

Surgical intervention is still the only possible radical method for the treatment of AMN. Specific interventions must be determined based on the size of tumor, location of the tumor, and its invasion and adhesion with abdominal organs (30). The present study followed the following principles: (a) appendectomy is carried out to the tumors located in the middle and distal end of the appendix and without invasion at the root of the appendix (applying to 20 cases); (b) if the tumor is located in the whole segment of the appendix or invades the root of the appendix, the appendectomy combined with partial cecectomy shall be performed (applying to 23 cases); (c) the appendectomy combined with right hemicolectomy is applied to those tumors that invade the cecum or the lower segment of the ascending colon (applying to 7 cases). In addition to the above principles, additional lymph node dissection was performed for those with tumor rupture during the intervention. Open appendectomy and laparoscopic appendectomy were applied to 37 patients (74.00%) and 13 patients (26.00%), respectively. In addition, 10 patients showed tumor rupture at presentation, and the tumor size was (6.85 ± 3.39) mm. Moreover, 12 cases of 50 patients with AMN were complicated with PMP. It suggested that the presence of PMP was associated with AMN. Ning et al. indicated that the occurrence of PMP was induced by LAMN accompanied by rectal cancer (31), and Reiter et al. revealed that the progression of PMP was related to LAMN (32). In this study, all patients were confirmed as AMN by pathological diagnosis after surgery, and LAMN accounted for 56.00%, and non-LAMN occupied 44.00%. Furthermore, we found that the incidence of tumor rupture and PMP in patients with non-LAMN was higher than that in patients with LAMN. CA19-9 is a tumor marker of pancreatic, gastric, and hepatobiliary malignancies. The high level of CA19-9 indicates that the lesion develops into malignancy, which is related to a poor prognosis (33). In our study, patients with non-LAMN presented a much higher level of preoperative serum CA19-9 than the patients with LAMN. These results showed that pTis, tumor rupture at presentation, the presence of PMP, and CA19-9 levels may be associated with the aggressiveness of AMN. To further analyze the risk factors of PMP in patients with AMN, we analyzed demographics, surgical management, and tumor characteristics of patients stratified by the presence and absence of PMP. It was revealed that age, tumor rupture at presentation, tumor size, and CA19-9 levels might be associated with the progression of PMP. We found that tumor rupture was a risk factor leading to PMP in patients with AMN, which was supported by other studies (34) indicating tumor rupture at presentation was the only factor significantly associated with PMP. The follow-up data (median: 19 months, ranging from 2 to 47.5 months) showed no significant difference in the OS between patients with non-LAMN and LAMN, and between patients with PMP and without PMP. However, the long-term effect needs to be further studied. It has been reported that no matter how benign or malignant AMN, once the tumor ruptures, it will significantly increase the postoperative recurrence rate and the risk of peritoneal implantation and metastasis, and affect the prognosis of patients (35). The present study found that 4 patients with previous PMP were diagnosed with PMP again and showed tumor rupture.

In conclusion, AMN is rare in clinics and lacks specific clinical manifestations. Clinicians should consider the possibility of AMN when the patient presents right lower abdominal pain, total abdominal pain, and upper abdominal pain. Abdominal color Doppler ultrasound and CT examination are helpful in making a clear diagnosis and guiding the treatment of AMN. Tumor rupture in AMN is a risk factor affecting the progression of PMP. However, further investigations with a large-scale sample size should be performed to provide a full understanding of the pathogenesis and progression of AMN.

## Data Availability Statement

The original contributions presented in the study are included in the article/supplementary material, further inquiries can be directed to the corresponding author.

## Ethics Statement

The studies involving human participants were reviewed and approved by Bozhou Hospital Affiliated to Anhui Medical University. The patients/participants provided their written informed consent to participate in this study.

## Author Contributions

ZBW conceived and designed the study and contributed to the writing of initial draft, and as well as review and editing. MMY contributed to study design, study validation, along with experimental resources, and software preparation. JYS assisted in writing, performed data analysis, and interpretation. ZPY was responsible for data verification and visualization. JP assisted in data analysis and verification. ZML contributed to funding acquisition and manuscript revision. All authors approved final manuscript.

## Funding

The study was supported by the National Natural Science Foundation of China (No. 81671886).

## Conflict of Interest

The authors declare that the research was conducted in the absence of any commercial or financial relationships that could be construed as a potential conflict of interest.

## Publisher's Note

All claims expressed in this article are solely those of the authors and do not necessarily represent those of their affiliated organizations, or those of the publisher, the editors and the reviewers. Any product that may be evaluated in this article, or claim that may be made by its manufacturer, is not guaranteed or endorsed by the publisher.
